# Super-enhancers: critical roles and therapeutic targets in hematologic malignancies

**DOI:** 10.1186/s13045-019-0757-y

**Published:** 2019-07-16

**Authors:** Yunlu Jia, Wee-Joo Chng, Jianbiao Zhou

**Affiliations:** 1 Cancer Science Institute of Singapore, National University of Singapore, 14 Medical Drive, Centre for Translational Medicine, Singapore, 117599 Republic of Singapore; 2 Department of Surgical Oncology, Sir Run Run Shaw Hospital, Zhejiang University, Hangzhou, 310016 Zhejiang China; 3 Department of Medicine, Yong Loo Lin School of Medicine, National University of Singapore, Singapore, 117597 Republic of Singapore; 4 Department of Hematology-Oncology, National University Cancer Institute of Singapore (NCIS), The National University Health System (NUHS), 1E, Kent Ridge Road, Singapore, 119228 Republic of Singapore

**Keywords:** Super-enhancers, Enhancer, Epigenetics, Hematologic malignancies, BET inhibitor, Combination therapy

## Abstract

Super-enhancers (SEs) in a broad range of human cell types are large clusters of enhancers with aberrant high levels of transcription factor binding, which are central to drive expression of genes in controlling cell identity and stimulating oncogenic transcription. Cancer cells acquire super-enhancers at oncogene and cancerous phenotype relies on these abnormal transcription propelled by SEs. Furthermore, specific inhibitors targeting SEs assembly and activation have offered potential targets for treating various tumors including hematological malignancies. Here, we first review the identification, functional significance of SEs. Next, we summarize recent findings of SEs and SE-driven gene regulation in normal hematopoiesis and hematologic malignancies. The importance and various modes of SE-mediated MYC oncogene amplification are illustrated. Finally, we highlight the progress of SEs as selective therapeutic targets in basic research and clinical trials. Some open questions regarding functional significance and future directions of targeting SEs in the clinic will be discussed too.

## Introduction

Enhancer is a class of regulatory DNA sequence that activates transcription of an associated gene from a distance of up to 1 Mbps (millions of base pairs) and independent of its orientation and location with respect to the transcription start sites (TSS) [1]. Enhancers contain clustered recognition sites for multiple transcription factors (TFs) and function as TFs binding platforms. TF binding to enhancers recruits coactivators, such as mediator (MED) complexes, CREB-binding protein (CBP), and p300. This binding also affects the three-dimensional structure of DNA, allowing the interaction between the activators and enhancers, the transcription factors, as well as the core promoter region and the RNA polymerase [2]. Accumulated evidence indicates that enhancers might be edited and maintained by epigenetic modifications such as the status of DNA methylation and histone modification and the differential recruitments of TFs during cell development and differentiation [3, 4]. Distinct functional states of enhancer landscape can be marked by the combinatorial patterns of histone modifications. Generally, functional enhancers are binding by monomethylation at lysine 4 (K4me1), acetylation at lysine 27 (K27 ac), and absent of trimethylation at lysine 4 (K4me3) of the histone H3 protein [5, 6]. Besides, active enhancers can generate enhancer-derived RNAs (eRNAs), and eRNA transcripts might contribute to enhancer-mediated target gene expression and function in transcriptional activation [7]. Notably, eRNAs transcription is positively concurrent with parameters of active enhancer elements, an enrichment of activated enhancer histone marks as H3K27ac, but decreased repressive mark H3K27me3 [8, 9].

**Fig. 1:**
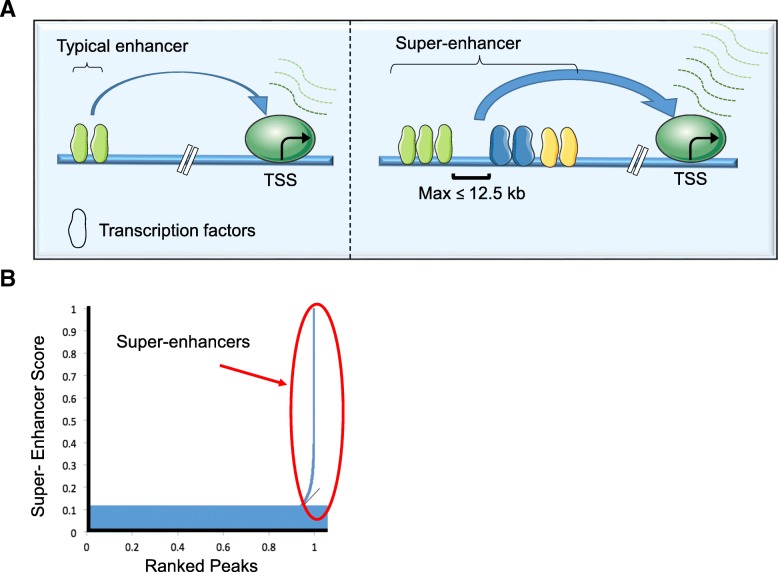
Schematic representation of typical enhancer versus super-enhancer. **a** A simplified comparison between typical enhancers and super-enhancers. Enhancers are orientation- and position-independent *cis*-acting regulatory elements distally located from the transcription start sites (TSS) [14, 15]. Enhancers are typically bound by multiple transcription factors to regulate gene expression outcomes. Regions of chromatin incorporating multiple enhancers, defined by ChIP-Seq (e.g., H3K27Ac, Med1, BRD4) within 12.5 kb, are referred to as super-enhancers. Super-enhancers are typically an order of magnitude larger than typical enhancers in size, have higher transcription factor density, and greater ability for transcriptional activation. **b** Enhancers are plotted in increasing order based on ChIP-Seq peak intensity. Super-enhancers are the population above the inflection point of the curve

In 2013, a number of papers proposed and experimentally demonstrated the new concept of super-enhancers (SEs) in mouse embryonic stem cells (mESCs) [10] and human cancers [11–13]. The identification of super-enhancer relies mainly on the chromatin immunoprecipitation (ChIP)-sequencing analysis using a combination of active enhancer marks (H3K27ac, H3K4me1), co-activators, and transcription factor profiles (especially cell-type defining TFs). In general, SEs are considered to be large clusters of regulatory elements (> 20 kb on average) with exceptionally higher (as compared to binding to typical enhancers) binding of transcriptional coactivators, such as mediator or EP300, or BRD4, or CDK7 [14], and have high potential to activate transcription of their target genes (Fig. 1). Bioinformatics algorithms via ROSE software locate genomic proximity for grouping elements to assign super-enhancer to a putative target gene. Indeed, the term “super-enhancer” has not been well-defined and the biological function is still controversial [15]. More specifically, it is unclear whether SE represents a simple assembly of regular enhancers or acts as an independent functional element through cooperative activities of its constituent enhancers [15]. SEs can drive the expression of genes that control and define cell identity and play important roles in cell type-specific biological processes [13]. Pluripotency genes, including OCT4, SOX2, and NANOG in mESCs, are all regulated by SEs [10]. During tumorigenesis, SEs-associated genes are key to the maintenance of cancer cell identity and promote oncogenic gene transcription [10, 12, 13]. Notably, SE-associated genes have significantly higher expression levels than genes under controlled by regular enhancers, which has been verified in a broad spectrum of cancers [16–18]. Cancer cells often rely on the SE-driven transcriptional program, which makes SEs and SEs-associated genes as promising therapeutic targets for further understanding cancer biology, clinical diagnosis, and therapy [12, 13, 19].

Other non-typical enhancers include stretch enhancers, shadow enhancers, and locus control regions (LCRs) [20]. Stretch enhancers are much larger than typical enhancers (≥ 3 kb vs median 0.8 kb), first identified by Francis Collins and colleagues [21]. SEs and stretch enhancers share some similarities: both of them are capable of driving cell type-specific gene expression and have high-density of TF binding [21, 22]. However, although the number of stretch enhancers exceeds that of SEs by an order of magnitude, SEs are transcriptionally more active and cell-type specific than stretch enhancers [23]. Another difference is that SEs are characterized based on the disproportionate abundance of mediator or H3K27ac signal, while the specific patterns of histone modifications or chromatin states of stretch enhancers are less emphasized [13, 21]. Some developmental control genes are regulated by more than one enhancer. In such circumstance, shadow enhancer refers to the most distal enhancer, while the relative proximal one is termed as primary enhancer [24, 25]. LCRs are described as DNA regions comprising a set of regulatory elements and activate associated genes expression. Indeed, SEs overlap with stretch enhancer and LCRs in mouse cell and human cancer cells [26]. A number of studies have documented that SEs and stretch enhancers overlap with the known LCR [13, 21, 22, 27].

Post-translational methylation of histone lysine modulates chromatin structure, thus playing an important role in transcriptional regulation. Epigenetic regulation of gene expression by histone methylation has similarity with SE-driving transcriptions. For example, as abovementioned, H3K4me1, enriched at the SE region, is catalyzed by the mixed lineage leukemia (MLL) family of methyltransferases (MLL2/3/4). Notably, genetic abnormalities involving the MLL gene has been widely identified in a broad range of acute leukemias and lymphomas (for comprehensive reviews, please refer to [28–31]). So, the aberrant MLL gene promotes the leukemogenic transcriptional program, which could be therapeutically targeted [32].

It has been well known that eukaryotic cells contain membraneless organelles, such as nucleolus, Cajal bodies, stress granules, nuclear speckles, and P bodies [33]. These biomolecular condensates are assembled through liquid-liquid phase separation and have an important function in transcriptional regulation and signaling transduction [34]. Interestingly, a recent study supported a model in which two key transcriptional coactivators of SEs, BRD4, and MED1 occupied discrete nuclear bodies and generated phase-separated condensates at sites of SE-driven transcription in mESCs [35]. SE condensates, transient liquid-like droplets, facilitated the compartmentalization and ensured robust transcription of cell-identity genes through the phase-separating properties of intrinsically disordered regions (IDRs) in TFs and cofactors [35]. This model explains well several key properties of SE, such as the robust regulation of multiple genes simultaneously, ultra-sensitive to perturbation. It is worth noting that this conceptual framework is newly developed, and more solid studies from different fields are required to confirm phase separation as a general principle for SE-driven transcriptional regulation. It is also interesting to investigate whether other types of enhancers as above-described also adopt phase separation model to regulate gene expression.

Considerable evidence indicates that transcriptional regulatory regions including SEs were disproportionately enriched with many disease-associated single nucleotide polymorphisms (SNPs), and the related gene dysregulation contributed to disease development and tumorigenesis [13]. For example, a single common causal SNP rs2168101 within a super-enhancer of LMO1 gene disrupted the binding sites of the transcription factor GATA, which subsequently contributed to neuroblastoma pathogenesis [36].

Hematologic malignancies are a group of cancers that affect blood and lymphatic system, including acute leukemias, chronic myeloid neoplasms, B and T/natural killer (NK) cell lymphomas, and multiple myeloma (MM) [37]. Hematological malignancies are often initiated by aberrant transcription such as alterations in enhancer landscapes, and consequently, aberrant super-enhancers result in the activation of abnormal gene transcription and lead to malignancies [38–40]. Notably, as SEs harbor large numbers of TFs binding sites, a small change in TF concentration causes significant changes in associated gene transcription, thus, disrupting this aberrant transcription is a promising approach for disease therapy. For example, SEs are associated with MYC and other key genes that have prominent roles in the biology of MM. Treatment of MM1.S cells with BRD4 inhibitor JQ1 inhibition leads to preferential loss of BRD4 at SEs and selective inhibition of the MYC oncogene [12]. Accumulating evidence demonstrates the importance of SEs in the regulation of key oncogenes in the pathogenesis of various hematologic malignancies [11, 41–43]. For example, oncogenic SEs contributing to malignancies have been reported in other hematologic malignancies such as lymphoma, T cell acute lymphoblastic leukemia (T-ALL), acute myeloid leukemia (AML), and chronic lymphocytic leukemia (CLL) [11, 42, 44–46].

In summary, most SEs are generated at key oncogenic drivers and are associated with genes that maintain hematopoietic identity in hematologic cancer cells, which highlight that generation of SEs is a common tumorigenic mechanism, raising the possibility for the therapeutic targeting of cancer cells and useful biomarker for disease diagnosis.

## Role of super-enhancer in normal hematopoiesis and cell transformation

Huang et al. reported that lineage-defining SEs are modulated pervasively and SEs-associated genes are crucial in normal hematopoiesis and hematopoietic cell differentiation [47]. Key hematopoietic genes such as ETV6, ERG, KIT, LMO2, MEIS1, and MED12 were associated with SEs function. MED12 interacted with P300 and located mostly at super-enhancers, and these SEs associated genes were relevant to cardinal hematopoietic transcription regulators for hematopoietic stem cells (HSPCs) function [48].

MYC is another key gene in the normal balance between HSC self-renewal and differentiation of HSCs [49]. MYC super-enhancer (SE) locates 1.7 Mb downstream to TSS of the MYC gene, and disruption of this region causes a significantly decreased MYC expression in HSCs and leads to increased differentiation-arrested multipotent progenitors. Importantly, this cluster of enhancers precisely controls MYC expression, which caused an accumulation of chromatin accessibility in human AML stem cell and also can be hijacked in malignancies [44, 50–52]. Recently, IKAROS and BRG1 were involved in the regulation of MYC SE during HSCs differentiation. Brg1 modulated chromatin accessibility to the MYC SE by allowing for a subset of B-lineage TFs (EBF1, IKAROS, and Pax5) binding to this super-enhancer region, subsequently activated MYC expression and prevents premature pre-B cell differentiation [53]. This MYC SE has been previously identified in AML where Brg1 is crucial to maintain MYC expression and promote the development of leukemia [52]. The MYC SE could be regulated by STAT5 and IKAROS by opposingly regulating histone acetylation and control pro-B cells proliferation and differentiation and B cell transformation. STAT5 binding to most of defined pro-B cell super-enhancer and increased high intensities of STAT5 binding to these super-enhancers is a prominent feature in B cell malignancies [54]. IKAROS regulates this developmental stage by positive and negative regulation of SEs with distinct lineage affiliations. Upon loss of IKAROS activity, “extralineage” TFs, including LMO2, LHX2, and the YAP–TEAD, are rapidly expressed and collaborate with native B cell TFs to define a de novo landscape of SE induce a gene expression program that provides pre-B cell with stem-epithelial cell properties before they become neoplasm [55]. Of clinical importance, high level of STAT5 or loss of IKAROS at these SE regions indicates poor prognosis in patients with pre-B acute lymphoblastic leukemia (ALL) [54, 55].

Similarly, in human T cell acute lymphoblastic leukemia (T-ALL), NOTCH MYC enhancer (N-Me) acts as a critical mediator of NOTCH1 induced MYC expression required for T cell development and T cell transformation. N-Me regulated MYC expression in hematopoietic stem cells and demonstrated a critical role of this regulatory element in the homeostasis of immature T cells. N-Me-mediated MYC upregulation was even more significant in leukemia initiation, where the loss of one and two copies of N-Me delayed and completely abrogated tumor development [37].

### Key mechanisms underlying the oncogenic super-enhancers formation

Pioneering studies in cancer cells showed that SEs are enriched at genes with known oncogenic function [10, 12, 13]. Broadly speaking, newly oncogenic super-enhancers are often acquired via (1) genomic elements rearrangements, (2) focal amplification, (3) small insertions and deletions, and (4) viral oncogenes.

### Genomic elements rearrangements

Chromosomal translocations are hallmarks of hematologic malignancies [56]. Enhancer hijacking could be one of the outcomes resulted from these translocations where unrelated SE regions are rearranged close to and activate some important oncogenes. MYC is one of such typical oncogene.

MYC proto-oncogene, located on chromosome 8q24, controls a plethora of target genes regulating the cell cycle, survival, differentiation, metabolism, and cell fate decision [57]. Chromosomal translocations involving MYC gene were identified in lymphoma in the early 1980s [58, 59], and aberrant MYC expression is frequently associated with disease progression and poor clinical outcome [49, 60, 61]. It has been now appreciated that super-enhancers related to MYC are frequently hyper-activated in a wide range of hematologic malignancies [12, 42, 61, 62].

Chromosomal translocation t(8;14), occurring about 15% of multiple myeloma (MM) cases [63], moves the strong immunoglobulin H (IgH) super-enhancer at chromosome 14 to the breakpoint at 8q24 near the MYC loci. The newly established super-enhancer profoundly increases the expression of MYC, causing an aggressive disease phenotype in myeloma patients [61]. In addition to this IgH-SE, MYC gene can hijack IgK-SE, IgL-SE, IgJ-SE, and non-Ig gene (like FOXO3, PRDM1, etc.) related SEs which are resulted from genomic rearrangements to drive its expression [12, 61, 62]. In diffuse large B cell lymphoma (DLBCL), the chromosomal translocation t(3;8)(q27;q24) creates the new fusion of the MYC with BCL6 gene, which results in MYC recruitment of BCL6-SE and subsequent activation of MYC expression to driving oncogenesis [64].

**Fig. 2:**
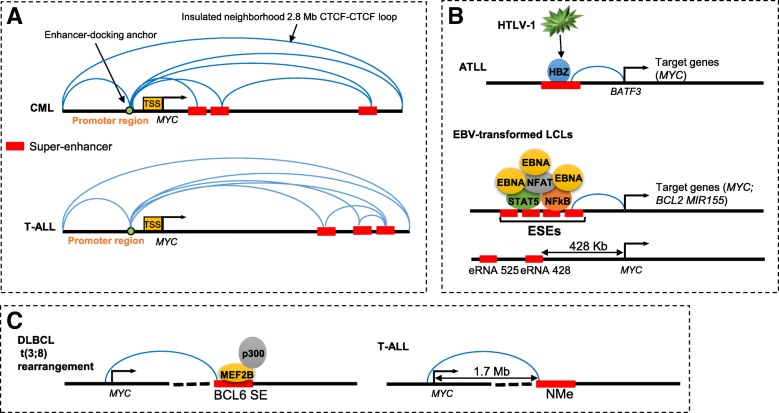
Different modes of super-enhancer-mediated MYC amplification. **a** In CML and T-ALL, super-enhancer interacts with a common and conserved CTCF binding site in MYC promoter [62, 76]. **b** In ATLL, HBZ (HTLV-I encoded transcription factor) binds to BATF3 super-enhancer and regulates the expression of BATF3 and its downstream target gene MYC (upper panel) [94]. ESEs cause upregulation of MYC (middle). eRNAs at ESEs − 428 and − 525 kb upstream of the MYC oncogene transcription start site affects MYC expression and cell growth (lower panel) [93]. **c** In DLBCL, a t(3;8)(q27;q24) chromosomal rearrangement directly links the MYC and BCL6 loci, resulting in MYC recruitment of BCL6 super-enhancers and subsequent activation of MYC expression (left panel) [64]. In T-ALL, NOTCH1 activates MYC expression via interaction of a long-range distal enhancer named N-Me (for NOTCH MYC enhancer) (right panel) [42]. HTLV-1, human lymphotropic virus type. EBV, Epstein-Barr virus. LCLs, lymphoblastoid cell lines; EBNA, Epstein-Barr virus nuclear antigen; ESEs, Epstein–Barr virus super-enhancers; eRNAs, enhancer RNAs; DLBCL, diffuse large B cell lymphoma; T-ALL, T cell acute lymphoblastic leukemia

Blastic plasmacytoid dendritic cell neoplasm (BPDCN) is a rare and an aggressive subtype of acute leukemia with strongly RUNX2 expression, and RUNX2 is also required for the tumor cell differentiation and migration [65, 66]. A pDC-specific RUNX2 super-enhancer is generated due to translocation (6;8), and activate the MYC expression and promote the proliferation of BPDCN [67]. Given its pivotal roles in cancer development, it is not surprising that MYC is one of the best and most studied oncogenes driven by SEs in hematological malignancies. The various modes of super-enhancer-mediated MYC amplification have been illustrated in Fig. 2. To a certain extent, we can consider that MYC-mediated transcriptional amplification or activation through SEs is an important hallmark of hematologic malignancies.

Translocations that put oncogenes under the control of SE can also be found in myeloid malignancies. Such “enhancer hijacking” occur in AML patients marked by inv(3) and t(3;3) karyotypes. AML with inv(3)/t(3;3) repositions a distal GATA2 enhancer and creates an ectopic SE for EVI1 aberrant activation and GATA2 functional haploinsufficiency and contributes to the cause of leukemogenesis [68].

## Focal amplification of super-enhancers

In addition to DNA rearrangements, aberrant oncogene expression can be driven by focal amplification of super-enhancers. For example, amplification of DNA segments was found within the super-enhancer of the MYC gene in lung cancer and endometrial carcinoma cell lines [69].

NOTCH1 is required for normal T cell development and is a key oncogenic driver in human T cell acute lymphoblastic leukemia (T-ALL) [70]. NOTCH1-binding sites are mainly found in SE region. NOTCH-associated SEs experienced extensive and dramatic NOTCH unloading, and reloading is spatially associated with robust NOTCH1 target genes. NOTCH1-SE interactions are important for the regulation of some key genes in NOTCH1-induced T-ALL cells [71]. MYC oncogene has been identified as a major target of NOTCH1 and involved in the pathogenesis of NOTCH-addicted T-ALL [72]. Recurrent focal amplification at chromosome 8q24 spanning a minimum conserved region of ~ 450 kb, which leads to a long-range distal enhancer named N-Me (NOTCH-bound MYC enhancer) formation and drives the transcriptional activation of NOTCH1 as an MYC downstream target gene [42]. As described previously, this super-enhancer is also essential for normal T cell differentiation. A more detailed analysis revealed that STAT5 and TLX1 co-bound and activated the N-Me and in the regulation MYC and BCL2 expression and promoted the development of T-ALL with NUP214-ABL1 fusion [73].

Similarly, in AML, the MYC SE located approximately 1.7 Mb downstream from the MYC TSS, which had five enhancer regions (E1–E5) and occupied by transcriptional co-factors such as BRD4 and BRG1. This region is contained in a number of focal DNA duplications [52]. Another study further confirmed that activated STAT5 strongly binding to E3 and E4 of MYC SE and contributed to BRD2 recruitment, resulting in transcriptional activations of MYC gene [74]. Additionally, the mechanisms underlying how specific super-enhancer loop long distances chromatin to interact with the MYC gene have been described in several studies. In Jurkat (T-ALL) and K562 (CML) cells, cancer-specific SE with diverse size and location has been proved to interact with a conserved CCCTC-binding zinc-finger protein (CTCF) binding site located 2 kb upstream of the MYC promoter and activate MYC expression [75]. A similar CTCF site interacts with MYC super-enhancer in MM [62]. A recent independent study in K562 cells revealed that MYC expression depends on the MYC CTCF site for the enhancer-promoter looping and transcriptional activation [76].

## DNA mutations and indels generating super-enhancers

In addition to large chromosomal alterations misregulating enhancer function, the DNA sequences comprising the enhancer itself can also be mutated to alter function in cancer cells.

In T-ALL, TAL1 functions as a regulatory complex with GATA3 and RUNX1 and coordinately co-regulate downstream target genes expression [77–79]. In a subset of T-ALL cases, the oncogenic function of TAL1 could be modulated by a super-enhancer within starting region of TAL1 locus. Short insertion mutations in a non-coding intergenic region of the TAL1 oncogene introduced binding motifs for the TF MYB. MYB, CBP, as well as RUNX1, GATA enhancers found in T-ALL cells, which suggests a broad function for MYB and CBP in super-enhancer initiation [80]. Larger topological domains (TAD) is a proposed self-interacting genomic region [81]. Recent studies have indicated that super-enhancer function is frequently confined within a loop connected by dimerization of the zinc finger protein CTCF and cohesion within TADs region [82–84]. In T-ALL, disruption of the DNA within CTCF binding sites resulted in insulation of the TAL1 and LMO2 in surrounding TADs. These genes are then activated by super-enhancers and cause the T cells transformation [85].

In normal HSCs, RUNX1 controls gene expression coordinately with other hematopoietic TFs such as GATA2 and TAL1 [86]. RUNX1 acts as a key component of the transcriptional regulatory circuitry in TAL1-addicted T-ALL and contributes to leukemogenesis. Super-enhancer analysis in Jurkat cells revealed that RUNX1 contains an exceptionally large SE region residing a previously described hematopoietic cell-specific enhancer [85]. Furthermore, GIMAP, a member of the GTP-binding superfamily, has been identified as a novel contributing factor to T-ALL leukemogenesis. TAL1, as well as RUNX1 and GATA3, binds at the SE site within the GIMAP gene cluster, resulting in its activation in T-ALL cells [87]. BCL2-modifying factor (BMF) protein contains single BH3 domain and opposes pro-survival BCL2 proteins, BCL-XL and BCL-W [88]. A meta-analysis of genome-wide association study (GWAS) revealed that the BMF gene locus on 15q15.1 carries CLL susceptibility [89]. In CLL, the polymorphisms on 15q15.1 risk locus generate super-enhancer for pro-apoptotic gene BMF and disrupt its RELA binding site, resulting in decreased BMF expression and its pro-apoptotic function [45].

## Super-enhancers activated by viral oncogenes

It is estimated that viral infections cause approximate 15 to 20% of all human tumors, and virus infection induces SEs formation at key genes involved in cell growth. Epstein-Barr virus (EBV) infection increases the risk of African Burkitt’s lymphoma, Hodgkin’s lymphoma, post-transplant lymphoproliferative disorder, and HIV-related lymphomas [90].

EBV super-enhancer (ESE) related genes, including MYC and BCL2 oncogenes, linked to lymphoblastoid cell lines (LCLs) survival. EBV oncoproteins such as EBNA2, 3A, 3C, and EBNALP and activated NF-kB subunits co-bind to hundreds of SEs to drive expression of some key pro-survival and anti-apoptotic genes, including MYC, MIR155, IKZF3, and BCL2, thus promoting LCL growth [91]. In EBV-infected B cells, multiple EBV nuclear antigens, including EBNA1, EBNA2, EBNA3, and EBNA-LP, control the expression of RUNX1 and RUNX3 through activation or repression of their associated SEs and contribute the transformation of B cell malignancies. Specifically, both of RUNX1 super-enhancer (− 139 kb to − 250 kb) and RUNX3 super-enhancer that are located − 97 kb from its TSS are activated by EBNA2 but inhibited by EBNA3B and EBNA3C [92]. Further study revealed that many eRNAs are transcribed from these Epstein–Barr virus super-enhancers (ESE). ESE eRNAs promote transcriptional activation of MYC oncogene, while silencing of MYC ESEs eRNA significantly inhibits cell growth [93]. The human lymphotropic virus type I (HTLV-I) retrovirus initiates adult T cell leukemia/lymphoma (ATLL). The viral transcription factor HBZ is expressed in all ATL cases. HBZ binds to a BATF3 super-enhancer and activates BATF3 and its downstream target MYC expression, thereby contributing to ATLL proliferation [94].

## Clinical application of super-enhancers

### Small molecule inhibitors targeting super-enhancers

Cancer cells hijack SEs to drive their oncogenic agenda, promoting survival and proliferation. This aberrant SE-driven transcriptional addition presents a therapeutic window and has been exploited for clinical anticancer treatment. Several classes of small molecule inhibitors have been developed for this purpose, and potent preclinical in vitro and in vivo efficacy has been observed. Some of these drugs are tested in different phases of clinical trials in hematologic malignancies and the preliminary results are encouraging. Drugs that target key components of super-enhancer, such as BRD4 and CDK7, provide a novel strategy for better cancer therapy.

## 1. BET (bromodomain and extra-terminal domain) protein inhibitors

BET family proteins, including BRD2, BRD3, BRD4, and BRDT, are epigenetic readers of histone acetylation, which play a key role in chromatin remodeling and transcriptional regulation. These proteins bind to acetylated chromatin and facilitate transcriptional activation. BRD4 was first identified as an interaction partner of the murine mediator co-activator complex and was subsequently shown to associate with mediator in a variety of human cell [95]. In 2010, JQ1 was the first BET inhibitor designed to target BET bromodomain (BD) [96].

JQ1 has a high affinity for bromodomains of all four members of BET family. After 1 year, BRD4 was identified as a therapeutic target in AML independently by whole-genome shRNA library screening method [97]. JQ1 exerts a robust anti-leukemic effect in vitro and in vivo through inhibition of MYC, as a result of BRD4 suppression. JQ1 competitively binds the two acetyl-lysine recognition bromodomains (BDI, BDII) of BRD4. Thus, JQ1 treatment displaces BRD4 preferably from histone protein and collapses super-enhancers. SE-driven oncogenes are more sensitive to JQ1 than other genes. One general mechanism for BET inhibitors to inhibit cancer growth is through the repression of cancer-specific SE-driven oncogenes. MYC is the exemplar of this category of oncogenes [97–100]. For example, treatment of JQ1 on MM cells led to decreased BRD4 binding at the SE region and subsequent repression of SE-associated target genes. Low concentrations of JQ1 have limited impact on global mRNA levels but it causes tremendous depletion of MYC and IRF4 mRNA, two pivotal oncogenes in the development of MM [12].

Soon after the discovery of JQ1, a series of different BET inhibitors has been emerged and tested in various types of cancers, as well as in other diseases. Targeting BRD4 has been demonstrated as a treatment option for AML and lymphomas. A novel potent and selective BET inhibitor (BI 894999) is highly active in AML cell lines, primary patient samples, and xenografts [101]. In AML xenografts, BI 894999 targeted SE-related oncogenes and other lineage-specific factors. Combination of CDK9 inhibitor with BI 894999 produces enhanced antitumor [101]. A structurally distinct BET inhibitor, INCB054329, selectively inhibits expression of bromodomain proteins and shows anti-proliferative function against hematologic malignancies [102]. OTX015 is another BET inhibitor targeting BRD2, BRD3, and BRD4, which strongly inhibits the cell growth in leukemia and lymphoma myeloma [103, 104]. Another BET inhibitor, I-BET151, was also found to antagonize SE-associated genes in AML [98].

In ATLL, BET inhibitors are profound toxic for ATLL cells in vitro and in vivo. JQ1 treatment depressed the eviction of BRD4 from interacting with BATF3 super-enhancer and inhibited BATF3 expression [89]. In diffuse large B cell lymphoma (DLBCL), JQ1 treatment limits the growth of DLBCLs and profoundly delayed tumor growth in two xenograft models. SEs are particularly sensitive to bromodomain inhibition, and JQ1-treated DLBCL cell lines showed significant transcriptional downregulation of MYC and E2F1 driven target genes and the significantly off-loading of BRD4 on promoters and enhancers [11].

Other bromodomains of the histone acetyltransferases CREBBP/EP300 are critical to sustaining the maintenance of super-enhancers and oncogene-driven proliferation in K562 cells. EP300 is extremely abundant at super-enhancers in K562 cells. Recent evidence suggests that a CREBBP/EP300 bromodomain inhibitor CBP30 disrupts the proper recruitment of CREBBP/EP300 to their binding sites and reduce GATA1- and MYC-driven transcription and consequently hold therapeutic potential [105].

Initial mechanistic studies uncovered that MYC, one of the master transcriptional regulators, was the target [12]. However, emerging evidence, including our data, reveals that inhibition of MYC does not correlate well with the drug efficacy [106]. These results suggest that MYC appears not the only target and the mechanism of BET inhibitor is not fully understood yet.

A novel and more potent small molecule pharmacological agent to target BRD4 has been described by the integration of BETi into a “proteolysis targeting chimera” (PROTAC) backbone [107, 108]. In Burkitt’s lymphoma (BL) cell lines, the PROTAC ARV-825 consists of OTX015 and pomalidomide has an apoptosis-inducing and antiproliferative function, which provide a better strategy for efficiently targeting BRD4 [108]. The PROTAC ARV-825 induces sustained apoptosis in CD34+ post-myeloproliferative neoplasms (MPN) secondary AML (sAML) cells. ARV-825 works synergistically with JAK inhibitor ruxolitinib and is still effective in ruxolitinib-resistant sAML cells [108]. Besides, PROTACs ARV-825 and ARV-771 lead to degradation of BET proteins in mantle cell lymphoma (MCL) cells. Co-treatment of ARV-771 with ibrutinib or CDK4/6 inhibitor palbociclib or the BCL2 antagonist venetoclax synergistically induces MCL cell apoptosis [109].

## 2. Cyclin-dependent kinase (CDK) inhibitors

Pol II is the central enzyme during protein-coding genes transcription initiation and elongation, which is regulated by a set of CDKs [110, 111]. CDK1 and CDK2 are representatives of cell cycle-related CDKs, while CDK7, CDK8, and CDK9 are a most transcriptional subfamily of CDKs. CDK7, cyclin H, and NMAT1 are parts of TFIIH complex, whose function is particularly crucial for transcription initiation and promoter pausing releasing. CDK7 phosphorylates the carboxyl-terminal domain of RPB1 (CTD) of RNA Pol II, rising transcription initiation. A novel CDK7 inhibitor, THZ1, has been discovered and characterized to have the ability to target the general transcriptional machinery and disrupt super-enhancers [112]. The anti-cancer propriety of THZ1 has been confirmed to target super-enhancers in neuroblastoma and lung cancer too [19, 113]. THZ1 treatment led to cell death in Jurkat T-ALL cells. SE-associated genes such as RUNX1, TAL1, and GATA3 are more sensitive to transcriptional inhibition by THZ1 exposure in T-ALL [42]. ATLL cells are particularly sensitive to THZ1 treatment, and THZ1 also inhibited the expression of SE-associated genes [41]. THZ1 was revealed as a covalent inhibitor of CDK7, which showed strong sensitivity to RUNX1- driven SE in T-ALL cell line [112]. THZ1 also presented selective inhibition of oncogenic super-enhancers in adult T cell leukemia cells [41].

### SE inhibitors targeting related pathway as cancer therapeutics

A novel approach to treat AML is by killing leukemic cells via activation of tumor suppressor p53, which could be achieved by casein kinase 1A1 (CKIα) inhibition [114, 115]. In a recent study, Minzel and colleagues developed a novel set of molecules targeting CKIα and CDK7/9. As oncogene-driving SEs are selective sensitive to CDK7/9 inhibition, thereby, these inhibitors block the transcription elongation of many SE-driven oncogenes and anti-proliferation in AML cells [116]. Mediator kinase inhibition with cortistatin A (CA) suppresses the growth of AML cells is part by suppressing STAT1 S727 phosphorylation, while STAT1 S727 phosphorylation tends to counteract CA-induced upregulation of some SE-associated genes [117–119].

### Small molecule inhibitors activating super-enhancers

#### 1. Cortistatin A (CA)

BRD4 and CDK7 are positive regulators of super-enhancer, while mediator-associated kinases cyclin-dependent kinase 8 (CDK8) and CDK19 have been identified as negative regulators of SE-associated genes in AML cells. One research revealed CA selectively inhibits mediator kinases, which has antiproliferative activity and significantly induces upregulation of SE-associated genes with tumor suppressive function, such as the TFs CEBPA, ETV6, and IRF [118].

#### 2. NCD38

Lysine-specific demethylase 1 (LSD1) regulates gene expression by affecting histone modifications and acts as an enhancer repressor. NCD38 is an LSD1 inhibitor, which inhibits the growth of leukemia cells by inducing leukocyte differentiation and activates approximately 500 SEs in leukemia cells. Upregulated genes with super-enhancer activation in erythroleukemia cells were mostly involved in myeloid differentiation. GIF1 and ERG are two key myeloid transcription factors driven by elevated SE activity. Importantly, the elevated SE activity occurs prior to the upregulation of GIF1 in response to NCD38 treatment. Overall, NCD38 derepresses super-enhancers of myeloid differentiation regulators that are abnormally silenced by LSD1, thus exerts an anti-leukemic effect through reversing the arrested differentiation programs [120]. This is the second example of fighting leukemia by activation of SE. Together with the abovementioned CA inhibitor, these data reinforce the concept of the existence of onco-supressive enhancers in cancers, which can be reactivated for therapeutic purpose.

## Super-enhancers predict drug sensitivity and identify response genes

SEs can be used as novel markers for predicting drug sensitivity and targeted therapy. For example, SEs mapping in AML patients revealed the presence of a super-enhancer at the retinoic acid receptor alpha (RARA) gene lead to high levels of RARA mRNA and sensitivity to SY-1425 (tamibarotene), a selective agonist of RARα. [43]. One important issue is to identify biomarkers that could predict potential responders for patient selection. Recently, HEXIM1 has been identified as a robust pharmacodynamic (PD) biomarker for BET inhibitor ABBV-075, MS417, and BI 894999 in xenograft tumor models [101, 121].

## Mutations in epigenetic modifiers impact on drug sensitivity

Alterations of epigenetic state driven by changes in chromatin regulators such as methylators, chromatin modifiers, and chromatin remodelers are a critical mechanism in the development of hematologic malignancies. Mutations in chromatin modifiers have significant effects on the treatment outcomes with SE inhibitors. Additional sex combs-like 1 (ASXL1) is a member of the polycomb group of proteins. Mutations in ASXL1 are frequently found in diverse myeloid malignancies and associated with worse outcome [122]. ASXL1 truncating protein acquires interaction with BRD4, while the wild-type (wt) ASXL1 protein does not. The interaction between ASXL1 with BRD4 results in a more open chromatin state, leading to a high sensitivity to BET inhibitors (EP-11313 and JQ1) [123]. These data suggest that BET inhibitors represent a promising therapeutic option for myeloid malignancies with ASXL1 truncation mutations.

DNMT3A is one of several epigenetic modifiers involved in most frequently mutated genes of hematologic malignancies. In AML, R882H mutations of DNMT3A leads to reduced DNA methylation at enhancer regions and activation of self-renewal gene programs, which is highly recurrent in AML and commonly related to poor risk disease [124–126]. JQ1 induced decreased cell viability and improved DNA damage in the AML cell line OCI-AML3 which harbored mutations in two genes, tNPM1 (exon-12) and DNMT3A (R882C) [127]. In the hematopoietic system, somatic mutations in the isocitrate dehydrogenase (IDH) genes IDH1 and IDH2 mutations are associated with loss of normal enzymatic function and cell differentiation block [128]. IDH2 mutant AMLs are more sensitive to BRD4 inhibition than IDH2-wt AMLs [129]. BRD4 by either BRD4-shRNAs or JQ1 triggers rapid differentiation and death of IDH2 mutant AML cells.

## BET inhibitor resistance

Resistance to BET inhibitors is mediated by diverse molecular mechanisms. Using different strategies, two groups reported back-to-back that WNT/β-catenin involves resistance to BET inhibitors. Rathert and colleagues demonstrated that silencing SUZ12, a component of the polycomb repressive complex 2 (PRC2), or DISP1 or DNMT3A can lead to AML cells resistance to JQ1 [130]. Inhibition of EZH2 and EED, the two other components of PRC2, achieved similar resistance phenotype. This resistance results from the activation and recruitment of WNT signaling constituents to compensate for BRD4 loss, a mechanism defined as “transcriptional plasticity.” In contradiction to the earlier findings that loss of PRC2 sensitizes brain tumor cells to BET inhibitors, ten-eleven translocation 2 (TET2), a dioxygenase, catalyzes DNA demethylation. TET2 mutations in AML contribute to hypermethylated DNA at enhancers, resulting in suppression of gene expression. TET2 mutation is a prognostic biomarker for inferior survival in de novo AML patients with normal karyotype (CN-AML). Leukemia stem cells (LSCs) or leukemia initiating cells (LICs) are a subpopulation of cells that acquire self-renewal function and sustain the disease [131]. It has been documented that AML LSCs are generally insensitive to conventional chemotherapy. Interestingly, Fong, et al. identified that resistance to BET inhibitors is mediated from LSCs in human and murine AML cells. Mechanistically, BRD4 is replaced with β-catenin at the MYC super-enhancer region in the BETi-resistant cells. Thus, MYC expression is driven by activated WNT/β-catenin signaling, instead of BRD4, rendering the resistance of cells to a different class of BET inhibitors. Indeed, pharmacological or genetic targeting Wnt/β-catenin signaling restores sensitivity to BET inhibitors [132]. Other novel mechanisms of BET inhibitor resistance have been uncovered in solid tumors. Speckle-type POZ protein (SPOP), a binding adaptor for the E3 ubiquitin ligase substrate, binds and catalyzes ubiquitination and proteasomal degradation of BET proteins. SPOP mutations in prostate cancer attenuate its binding ability to BET proteins and decreased proteasomal degradation. Hence, SPOP mutant prostate cancers are resistant to BET inhibitor owing to having more stabilized BET proteins than SPOP-wt cancers [133, 134]. BRD4 is a substrate of deubiquitinating enzyme 3 (DUB3). Upregulation of DUB3 induced by nuclear receptor corepressor 2 (NCOR2) gene deletion in prostate cancer leads to BET inhibitor resistance resulting from elevated BRD4 [135], which converges with SPOP mutation. Resistance rooted from SPOP mutations and NCOR2 deficiency appears cancer-type specific to prostate cancer because SPOP mutations in endometrial cancer sensitize BET inhibitor, an effect converse in prostate cancer. Furthermore, a similar resistance mechanism has not been identified in hematologic malignancies.

## Clinical development of BET inhibitors

Novel BET inhibitors have moved quickly into clinical trials since the discovery of JQ1 in 2010. The report of the first clinical trial of EBT inhibitor MK-8628/OTX015 was published in 2016 by a group of European and Canadian oncologists [103, 104]. In this phase I dose-escalation study, complete response or partial response was achieved in several patients with advanced AML or lymphoma. The common side effects include hematologic events (thrombocytopenia, neutropenia, and anemia) and others such as diarrhea, fatigue, and nausea. Severe grade 3–4 toxicity is infrequent [103, 104]. However, it is worthy of noting that the downregulation of MYC mRNA protein is not correlated with drug sensitivity. Thus, MYC could be used as a predictive biomarker for response. Preliminary analysis of CPI-0610 in refractory or relapsed lymphoma trial revealed that anti-lymphoma efficacy has been confirmed in 15 out of 64 patients (23.4%) enrolled [136]. Currently, there are 10 BET inhibitors in phase 1/2 clinical trials for hematologic malignancies (Table 1). Overall, these early results from these clinical trials show modest response and manageable toxicity. A first-in-class, second-generation BET inhibitor ABBV-744, which selectively targets the BDII domain of BET proteins, has been developed. Compared to those first-generation BET inhibitors, the potent anti-cancer effect of ABBV-744 has been observed mainly in AML and androgen receptor-positive prostate cancer models, but with significantly improved oral bioavailability and tolerability [137]. We shall wait to see if this second-generation of BET inhibitor can produce better response and reduced drug toxicities in the clinical trials.

**Table 1:** The list of BET inhibitors in clinical trials in hematologic malignancies

Drug name	Other name	Trade name	Structure	Class (target)	Disease	Combination	Phase	Status	Company
FT-1101		NA	Unrelated to JQ1	Pan BET inhibitor	R/R: AML, MDS, NHL	Azacitidine	1	Recruiting	Forma Therapeutics
RO6870810	TEN-010	NA	An analog of JQ1	Pan BET inhibitor	R/R: MM	Daratumumab	1b	Recruiting	Hoffmann-La Roche
	RG-6146, JQ2				R/R: AML, MDS		1	Recruitment completed	
CPI-0610*		NA	Benzoisoxazoloazepine	2-time potent for BRD4 than BRD2, 3, T	R/R: MM		1	Recruitment completed	Constellation Pharmaceuticals
				6-time potent for BDII than BDI	R/R: DLBCL, follicular lymphoma		1	Preliminary analysis released	
					R/R: AML, MDS		1/2	Recruiting	
					Lymphoma		1	Active, not recruiting	
GSK525762	I-BET762	Molibresib	Benzodiazepine	Pan BET inhibitor	R/R: AML, MDS, NHL	None	1/2	Recruiting	GlaxoSmithKline
INCB054329			Diazaacenaphthylen	More potent to BRD2, 3, 4 than BRDT	Advanced hematologic malignancies	None	1/2	Recruitment completed	Incyte Corporation
BMS-986158		NA	Carboline based		Advanced hematologic malignancies	Nivolumab	1/2a	Recruiting	Bristol-Myers Squibb
MK-8628*	OTX-015	Birabresib	Methyltriazolodiazepines	Pan BET inhibitor	Hematologic malignancies		1b	Reported	Merck Sharp & Dohme Corp
					De novo and sAML, DLBCL		1	Active, not recruiting	
ABBV-075		Mivebresib	Ethanesulfonamide	More potent to BRD2,4,T than BRD3	AML, MM, NHL	Venetoclax	1	Recruiting	AbbVie Inc
ABBV-744		NA		highly BDII-selective	R/R: AML		1	Recruiting	AbbVie Inc
AZD5153		NA	Triazolopyridazine	Bivalent inhibitor	R/R: lymphoma		1	Recruiting	AstraZeneca

*trial results released. R/R: relapsed/refractory

## Promising combination therapies with super-enhancer inhibitors

Monotherapy with a targeted agent for a prolonged period often leads to the development of drug resistance [138]. A large body of studies in the literature suggests that BET inhibitors could achieve synergy and overcome acquired drug resistance when combined with chemotherapy or other epigenetic agents or kinase inhibitors or antibody therapy. We summary these studies in Table 2 and expand discuss for selective studies.

**Table 2:** Summary of combination studies using BET inhibitors in hematologic malignancies

Disease	Combination	Effect	Reference
**Lymphoma**	**OTX015**, mTOR inhibitor everolimus, BTK inhibitors ibrutinib, HDAC inhibitor vorinostat, and anti-CD20 monoclonal antibody rituximab	Synergic anti-lymphoma activity *in vitro* and *in vivo*	Boi M, et al [154]; Gaudio E, et al [155].
Diffuse large B cell lymphoma cell lines and xenograft mouse model			
Activated B-cell-like (ABC) DLBCL cells and xenograft mouse model	**JQ1**, BTK kinase inhibitor rbrutinib	Reduce IκB kinase activity and inhibit cells proliferation	Ceribelli M, et al [151]
Lymphoma-derived cell lines	**BAY 1238097**, EZH2 inhibitor DZNep, GSK-126, everolimus and ibrutinib	Synergistic cytotoxicity *in vitro*	Bernasconi E, et al [156]
B- cell lymphoma xenograft mouse model	**RVX2135**, ATR inhibitor AZ20	Enhanced sensitivity to ATR inhibitor *in vitro* and in mouse models of B-cell lymphoma, and improve survival	Muralidharan, et al.
Myc-transgenic mice lymphoma models	**RVX2135**, HDAC inhibitor panobinostat, vorinostat	Inhibit proliferation and induce apoptosis of lymphoma cells	Bhadury, et al [141]
Human lymphoma B-cell lines	**JQ1**, rituximab	JQ1 Increase Rituximab sensitivity in lymphoma cells with CYCLON and MYC expression	Emadali A, et al [152]
Primary effusion lymphoma (PEL) cell lines and orthotopic xenograft model	**JQ1**, lenalidomide	Synergistic cytotoxicity in PEL cell lines an increased the survival of PEL bearing NOD–SCID mice	Gopalakrishnan, et al [157]
Mantle cell lymphoma cell lines and mice models	**CPI203**, lenalidomide	Synergistic antitumor activity in bortezomib-resistant mantle cell lymphoma	Moros A, et al [153]
**Leukemia**	**JQ1**, cytarabine (ARA-C)	Synergistic anti-leukemic effects	Herrmann H, et al [146]
Acute myeloid leukemia (AML) cell lines HL60 and KG1			
Patient-derived AML cells and mice model	**JQ1**, JAKi ruxolitinib, HSP90i AUY922	Inhibit growth and induce apoptosis of patient-derived AML cells, and improved survival of mice engrafted with AML cells; Enhanced sensitivity to ruxolitinib-resistant AML cells.	Saenz DT, et al [158]
AML cell lines, primary patient samples, and xenograft models	**BI 894999**, CDK9 Inhibitors flavopiridol, LDC000067	Synergistic antitumor effects and lead to rapid induction of apoptosis *in vitro* and *in vivo*.	Gerlach D, et al [101]
AML cells expressing FLT3-ITD and TKI-resistant cell lines	**JQ1**, FLT3 inhibitor ponatinib, AC220, panobinostat	Inhibit growth and induce apoptosis of human AML cells; Synergistic induction of apoptosis in TKI-resistant cells	Fiskus W, et al [140]
AML xenograft mouse model	**ABBV-075**, Bcl-2 inhibitor venetoclax, hypomethylating agent azacitadine, proteasome inhibitor bortezomib	augments the activities of venetoclax, azacitidine, in xenograft models of AML.	Bui MH, et al [144]
Human AML xenograft models	**INCB054329** and LSD1 Inhibitor INCB059872	Enhanced myeloid differentiation and apoptosis in human AML cell lines	Liu X, et al [145]
Human T-cell acute lymphoblastic leukemia (T-ALL) cells	**JQ1** and CK2 inhibitor CX-4945	Induce apoptosis in human T-ALL cells,	Lian H, et al [149]
Primary Acute lymphoblastic leukaemia (ALL) cases, ALL cell lines and ALL xenograft models	**JQ1** and dexamethasone	JQ1 sensitized ALL cells to dexamethasone, and reduced subcutaneous tumor growth in ALL xenograft models.	Da Costa D, et al [147]
**Myeloma**	**INCB054329** and JAK inhibitors (ruxolitinib or itacitinib)	Inhibit myeloma cell growth *in vitro* and *in vivo*	Stubbs MC, et al [102]
Multiple myeloma (MM) models			
*In vitro* and *in vivo* models of MM	**CPI203** and lenalidomide/dexamethasone	Improve therapy response in relapsed/refractory patients with MM	Díaz T, et al [159]
bortezomib and melphalan resistant MM cell lines and patients sample	**CPI203** and bortezomib	enhanced apoptosis and anti-proliferative effects	Siegel M B, et al [160]

Compounds in boldface are BET inhibitors

In multiple DLBCL cell lines, the BET inhibitor OTX015 exerted significant synergy in vitro with a number of standard anti-lymphoma agents, including lenalidomide, rituximab, decitabine, everolimus, ibrutinib, idelalisib, and vorinostat [139]. Moreover, a BET inhibitor BI894999 exerted synergistic tumor growth delay with the CDK9 inhibitors alvocidib and LDC000067 in AML xenografts [101]. In AML with FLT3-ITD expression, JQ1 and the FLT3 tyrosine kinase inhibitor (TKI) ponatinib or AC220 synergistically induced cell apoptosis [140].

Combining BET inhibitors and epigenetic directed therapies is another promising area of synergy. Combinations of BET inhibitors and HDAC inhibitors improve cytotoxicity in multiple disease models. The synergistic anti-tumor effect with the combination of RVX2135 and HDAC inhibitors (panobinostat/vorinostat) was reported in mice lymphoma xenografts [141], AML cells [142], and DCBCL [143–145].

BET inhibitors can be combined with chemotherapy for a better anti-cancer response. JQ1 exerted synergistically with cytarabine in the treatment of AML [146]. Acute lymphoblastic leukemia is more responsive to BET inhibitors when co-treatment with dexamethasone [147]. Co-treatment with BETi and ruxolitinib synergistically induced cell apoptosis and improved survival of cultured and PD sAML cells [148].

The combination CK2 inhibitor CX-4945 synergizes with JQ1 against human T-ALL cells by targeting NOTCH1 signaling [149]. CX-4945 induces pro-apoptotic unfolded protein response (UPR) in T-ALL cells, while JQ1 downregulates MYC that normally activates pro-survival UPR. Hence, CX-4945 and JQ1 may also synergistically kill T-ALL cells by enabling the switch of pro-survival to pro-apoptotic UPR. JQ1 exhibit a strong efficacy in treating DLBCL model Ly1 with JQ1 [150] and synergistic effect with ibrutinib was also reported recently [151]. Another work reported that combining JQ1 with rituximab increased the sensitivity of human lymphoma B cells to this drug [152]. Mantle cell lymphoma cell lines exhibit high sensitivity to the BET inhibitor CPI203, while a combination of CPI203 with lenalidomide displays synergistic effects and leads to an apoptotic response in the bortezomib-resistant REC-1 model [153].

## Conclusive remarks

We have witnessed an exceptional expansion of super-enhancer research in oncology and beyond. It is now apparent that super-enhancers play critical roles in transcriptional regulation, which have the oncogenic capacity or onco-supressive potential in a context-dependent manner. The discovery that JQ1 can target SEs has led to the development of several first-generation and second-generation BET inhibitors. In the following years, we shall have more comprehensive knowledge of their clinical efficacy and toxicity when more BET inhibitor trials are mature. Currently, CDK7 inhibitors, SY-1365, and CT7001 are evaluated in clinical trials for advanced solid tumors and, so far, not in trials for hematologic malignancies (ClinicalTrials.gov). Similarly, we are waiting for the use of drugs activating onco-supressive SEs in clinical trials too.

Currently, we primarily utilize ChIP-seq data and bioinformatics algorithm to identify genomic proximity for grouping elements to assign super-enhancer to target genes. However, knowledge of intrinsic properties of super-enhancer and how exactly they interact with target gene in three-dimensional (3D) genome native environment remains insubstantial. An integrated approach including chromosome conformation capture carbon copy (5C) and functional screening is needed to carefully interrogate these fundamental questions. Recent years have witnessed a rapid advance in technologies and assay developments, which offer novel insights into the function and biophysical formation (phase separation) of SE, as well as the regulatory mechanisms of the targeted gene. The assay for transposase-accessible chromatin with high-throughput sequencing (ATAC-Seq) can be performed on fewer than a thousand of cells or even on single cell level (10x genomic Inc., Pleasanton, CA, USA) to map open chromatin and nucleosome positions [154]. Cleavage Under Targets and Tagmentation (CUT&Tag), evolved from CUT&RUN, is another novel assay for in situ profiling variety of chromatin components at high-resolution without cross-linking [155, 156]. Hopefully, the emergence of integrated new technologies including CRISPR genome editing tool, single-cell sequencing technology in couple with ChIP-seq, ATAC-seq, and CUT&Tag, will uncover novel insight into the roles of super-enhancers regulating transcriptional machinery and oncogenesis, as well as the drug development for targeting super-enhancers.

## Data Availability

The datasets supporting the conclusions of this article are included within the article.

## Abbreviations

5C: Chromosome conformation capture carbon copy
AML: Acute myeloid leukemia
ATAC-seq: The assay for transposase-accessible chromatin with high-throughput sequencing
CR: Complete response
CUT&Tag: Cleavage Under Targets and Tagmentation
DLBCL: Diffuse large B cell lymphoma
EBNA: Epstein-Barr virus nuclear antigen
EBV: Epstein-Barr virus
eRNAs: Enhancer RNAs
ESE: Epstein–Barr virus super-enhancers
FL: Follicular lymphoma
HL: Hodgkin lymphoma
HTLV-1: Human lymphotropic virus type-1
IDRs: Intrinsically disordered regions
LCLs: Lymphoblastoid cell lines
LSCs: Leukemia stem cells
MDS: Myelodysplastic syndrome
MM: Multiple myeloma
NHL: Non-Hodgkin lymphoma
PROTAC: Proteolysis targeting chimera
R/R: Relapsed/refractory
sAML: AML secondary to MDS
SE: kSuper-enhancer
T-ALL: T cell acute lymphoblastic leukemia
